# Early Eating Behaviours and Food Acceptance Revisited: Breastfeeding and Introduction of Complementary Foods as Predictive of Food Acceptance

**DOI:** 10.1007/s13679-016-0202-2

**Published:** 2016-03-08

**Authors:** Gillian Harris, Helen Coulthard

**Affiliations:** School of Psychology, University of Birmingham, Edgbaston, Birmingham, B15 2TT UK; Division of Psychology, De Montfort University, Leicester, LE1 9BH UK

**Keywords:** Infant, Child, Food acceptance, Taste exposure, Breastfeeding, Fruit and vegetables

## Abstract

Current dietary advice for children is that they should eat at least five portions of fruit and vegetables a day (Department of Health. National Diet and Nutrition Survey, 2014). However, many parents report that children are reluctant to eat vegetables and often fail to comply with the five-a-day rule. In fact, in surveys carried out in areas in the UK, the number of children eating according to the five-a-day rule has been found to be as low as 16 % (Cockroft et al. Public Health Nutr 8(7):861–69, 2005). This narrative review looks at those factors which contribute to food acceptance, especially fruit and vegetables, and how acceptance might be enhanced to contribute to a wider dietary range in infancy and later childhood. The questions we address are whether the range of foods accepted is determined by the following: innate predispositions interacting with early experience with taste and textures, sensitive periods in infancy for introduction, breastfeeding and the pattern of introduction of complementary foods. Our conclusions are that all of these factors affect dietary range, and that both breastfeeding and the timely introduction of complementary foods predict subsequent food acceptance.

## Introduction

### Early Food and Taste Acceptance

Infants are born with specific taste preferences and aversions; however, specific food preferences cannot be hardwired; humankind needs to be flexible about which foods can be accepted because different cultures depend upon a wide range of foodstuffs. It would therefore be useful for infants to rapidly accept predominant tastes that define the foods of their culture or subculture so that they are able learn to like the food available in their environment. In general, the foods we learn to like in infancy and early childhood do predict those that we eat in later childhood and adulthood [2]. However, although it would seem that these preferences are mostly learned postnatally, it would also seem that there are innate preferences which ensure acceptance of sweet, smooth and high energy density foods [3, 4•] which predict good sources of energy and which are easily consumed.

In addition, there are marked differences in the willingness to accept food tastes and textures, and to try new foods that have not already been introduced to the diet, both in children and adults. To some extent, most people are ‘fussy’ in that they have a few foods that they will not eat, and most are reluctant to eat very novel foods from different cultures. In the UK, from clinical experience, the foods that are usually found aversive by adults are those of difficult texture, shell fish, bananas and mushrooms, or taste and smell, such as olives and fish.

This ‘fussiness’, which is a rather poorly defined term, is however more apparent in children. There is also a key stage in development during which children show extreme new food refusal, the neophobic stage, and it is not always clear how distinct ‘fussiness’ is from neophobia [5]. This stage which peaks at around the age of 20 months, is more extreme in some children than in others [6] and gradually fades away by the age of 5 to 8 years [7]. There is then an interaction between the innate reluctance of some children to accept tastes, textures and new foods and the effect of early exposure to new tastes and textures. A child might not accept a food because of innate predisposition or because they have not been given that food during a possible ‘sensitive’ period for introduction and familiarisation.

Research studies in this area report on different methodologies, some looking at intake of a food in early infancy before the neophobic stage has been reached [8, 9, 10••, 11•], others looking at acceptance of a new food during the neophobic stage [12, 13], and further studies, usually longitudinal, looking at dietary range in older children [14, 15]. The research studies reviewed here, therefore, cover a range of methodologies and look at short term or long term influences on food acceptance and dietary range and cover acceptance of and preference for odours, tastes, textures and foods.

### Early Defining Exposure Experiences

#### Amniotic Fluid

There is some evidence that the experience of amniotic fluid, in turn affected by maternal diet, determines some preferences observed in the new born infant, but these observed preferences are for a specific odour. Infants orient towards the odour of flavours experienced in amniotic fluid of foods eaten by their pregnant mothers [16]. Not all food flavours can pass through to this medium, and those that do tend to be strong and rather have idiosyncratic tastes, such as garlic or anise [16–18]. Prenatal learning through exposure to the uterine environment occurs, but the demonstrated preference is not one of intake but of an orienting response; and, reported influence on subsequent food acceptance in the infant is not well founded. The one study that has found an association with later preference, for example prenatal garlic exposure predicting consumption of a gratin containing garlic at 8–9 years, does not control for the interim exposure period and, therefore, does not provide evidence for the long term effects of prenatal flavour exposure [19].

### Birth

The infant is born with a preference for a sweet taste [3, 20] and with a relatively neutral or positive response to salt and sour tastes, and possibly to umami, depending on the concentration used when testing. There is, however, a distinct aversive response to a bitter taste [3, 21]. This preference is thought to be adaptive in that sweet tastes are usually associated with good sources of energy [22]. The aversion to a bitter taste is adaptive in that this taste is often associated with toxicity [23], and this is why many plants (or green vegetables) have developed a bitter taste, to prevent being eaten by mammals. There is, however, variation in the extent of bitter taste aversion. Bitter ‘supertasters’ can be found in both adult and child populations [24, 25], which makes the exposure to and acceptance of such tastes especially difficult. Supertasters have been found to have a reduced liking for cruciferous and *Brassica* vegetables, such as broccoli [26]. There is also a possibly genetically determined response to other tastes such as geosmin, the earthy quality present in certain foods such as beetroot and mushroom [27].

Heritability in the acceptance and rejection of foods has also been observed, both in a general neophobic response [28] and of rejection of specific foods (meat, fish, fruit and vegetables) but not fatty foods of smooth texture such as yoghurts. However, this rejection could be one of texture, rather than taste [29]. It has been reported that infants in the transition to solid foods do not accept foods such as leafy vegetables and sliced meat well because of the texture of these foods [30].

### Early Milk Feeding

#### Formulae

Some flavour preferences might be learned from the intra-uterine environment, but they are also learned during the early stage of milk feeding; modified by exposure and to some extent predicting subsequent acceptance of foods. However, whilst this learned preference does not seem to be for specific foods (or flavours) eaten by the mother whilst breastfeeding, specific taste modification has been reported in formula-fed infants who have been exposed to bitter hydrolysate formulae, and this modification of taste acceptance is most marked if started shortly after birth [31]. This easy acceptance of a bitter tasting formula after early exposure continues into later childhood [32] and, to some extent, generalises to other similar tastes. Children fed with bitter hydrolysates during infancy preferred sour flavoured juices at 4–5 years [33]. However, the preference would seem to be context specific, a higher intake of a bitter food (broccoli) rather than sweet food (carrot) was not found in infants fed with vegetable hydrolysates compared with those fed normal formula [34].

### Breastfeeding

#### Learned Preference for Specific Foods

The transmission of taste compounds from the mother’s diet through breast milk to the infant has been observed, but can vary widely from mother to mother, differ according to the food eaten, and the compounds are transferred in relatively small amounts. The change to the taste of the mother’s milk is therefore likely to be subtle and variable [35]. Some specific and rather idiosyncratic transmission has been noted, such as that of garlic, caraway, cigarettes and alcohol [36–39]. This changeable nature of breast milk does seem to facilitate the acceptance of complementary foods when these are introduced. However, research does not support the idea that increased acceptance of specific pureed food fed to infants is linked to specific foods in the maternal diet [39]. Menella, Jagnow and Beauchamp [40] found ratings of greater enjoyment of a target food (carrot) but not an increase in intake between the infants of those mothers who were exposed to carrot and those who were not. Similarly, pureed green beans and peaches were given to infants of mothers who had either breast fed, were still breastfeeding or were formula fed [41]. Infants who were breastfed ate more of the peaches, and mothers of the breast fed infants ate more fruit during the week prior to testing, but not peaches specifically. There was no difference in intake of green beans between formula fed (FF) or breast fed (BF) infants. Infants increased their intake of green beans after a period of 8 days of exposure, regardless of whether they were FF or BF, suggesting that exposure to the actual foods themselves is a much more robust effect.

#### Generalised Food Acceptance

Therefore, although breast feeding would seem to confer some advantage over formula feeding in subsequent food acceptance, the effect is more that of the acceptance of taste change or taste variety. Consumption of a specific food does not predict a preference for that food rather than an isolated taste or flavour, but it is more that the taste of breast milk fluctuates according to changes in maternal diet, whereas infant formula milk does not vary in taste. The enhanced acceptance is therefore based on a generalisation effect, the greater the varied experience of tastes then the better the acceptance of a new taste; a generalisation effect also observed throughout the introduction of complementary foods (ICF). What is common to each of these studies looking at the effect of breastfeeding is that each study includes some exposure to complementary foods fed to infants via the spoon, and that even infants who are formula fed respond quickly to this exposure in the ICF period. Infants exposed to the flavour of caraway through breast milk showed a subsequent higher intake of caraway-flavoured puree, but this heightened preference in comparison with formula fed infants was no longer evident after a 10-day exposure period for all infants [42].

#### Long-Term Effects of BreastFeeding

A beneficial effect of breastfeeding has been noted in studies looking at food acceptance in older children and later infancy [5, 43]; however, it is not always clear which intervening factors might be operating and whether or not factors such as maternal SES and early feeding practice have been controlled for [10••, 15]. Both parental educational levels and breastfeeding predict the higher consumption of vegetables [44]. Higher SES mothers were more likely to have foods such as fruit and vegetables in the house, certainly if they are eating these themselves [45] the infant will therefore be exposed to the sight and smell of the foods, as well as the taste via breast milk, and these in turn will affect food intake [46, 47•]. It could also be that higher SES mothers who breastfeed are more likely to give the infant home-prepared foods rather than to rely on commercial baby food, and this trend in itself has been shown to predict subsequent fruit and vegetable intake in older children [14, 48, 49].

### Age of/ Introduction of Complementary Foods

#### Tastants Added to Foods

There is a clear learned acceptance of a specific taste in first foods given to an infant. In a sample of 6-month-old infants, who had already been started on solids, there was a relationship between the infant’s experience of a taste (salt) and their acceptance of the taste in a bland rice base [50]. This acceptance and preference was quickly learned and was higher in infants aged 16–17 weeks than in infants 18–25 weeks [51]. Single tastes are therefore rapidly accepted; real foods, however, have a more complex combination of flavours, and so we cannot assume that infants learn to accept more complex tastes as rapidly, nor do we know whether preference in complex tastes would be for the predominant taste or for all of the taste compounds.

#### Preference for Specific Foods

The advantage of exposure to foods rather than flavours which pass through breast milk is that the tastes that are experienced are usually in the context and combination that will be carried on into adulthood. Although this may not be true if the infant is fed a diet predominately comprising commercial baby food, in which tastes are often masked by other more acceptable sweet tastes [14]. There has been one study [8] which has attempted to bridge this gap between milk flavour and first food acceptance. Mothers of infants with an average age of 5 months were asked to feed their infant expressed breast or infant formula with added vegetable puree for 12 days, baby rice with the added vegetable puree for a further 12 days, followed by 11 days of exposure to the vegetable puree alone. At follow-up, vegetable puree intake was measured and there was an effect of exposure, the intervention group showed increased vegetable intake specific to those vegetables introduced. However, the infants were not assessed at the end of the milk feeding intervention so this effect could be merely due to the early experience of vegetable puree and rice.

In a similar study, [52] foods were introduced to bottle fed infants at a mean age of 4 months and tested 3 weeks later. After a 9-day exposure period during which infants were either fed carrots, potatoes or variety of vegetables, infants ate more carrots after exposure to carrots. When chicken was introduced as a new taste, infants in the variety group ate more than the other groups. Variety of early exposure does seem to influence the acceptance of new foods. In both of these studies, however, it was noted that infants always seem to prefer vegetables such as carrots, which have an inherently sweet taste, to green beans or potatoes. It would seem then that it is relatively easy to induce a food preference with repeated exposures, where there is a similarity to an innate taste preference, or an already accepted food, [8, 9, 39, 52].

#### Generalisation Effect

Two studies have looked specifically at generalisation effects; that is whether new foods are more likely to be accepted if a variety of foods are offered initially. In the first study, infants (mean age 5.2 months) were exposed to a single vegetable, a variety of vegetables with daily change, and a variety with change every 3 days, for 9 days. Where the food had been rotated daily, infants showed an enhanced acceptance of a new food (zucchini-tomato, peas, meat and fish) [10••]. However, this finding was not replicated in a recent study [11•] looking at early and late introduction of vegetables within the 4–6-month period. Acceptance of a novel vegetable was measured after a 9 day exposure period in two groups of infants. During the exposure period one group was given a single vegetable, one group was given a variety pack of three vegetables. Although there was no main effect of vegetable variety on new food acceptance, there was an interaction between age of introduction and variety; acceptance at the later age (5.5–6 months) was better if a variety of vegetables rather than a single vegetable had been given. This suggests a sensitive period for the acceptance of new tastes, similar to previous taste studies [50], early within the introductory period.

#### Long-Term Effects

Long-term effects of the timing and type of complementary foods introduced have been reported in various studies, looking at children of different ages. More frequent acceptance of new foods during the neophobic period has been reported in those children who were introduced to complementary food earlier within the usual period of introduction [12] (4–6 months commonly reported in the UK [1]. And, the earlier the age that children had been introduced to fruit and vegetables (mean age of introduction for fruit 4.8 months and for vegetables 6.2 months) the greater the child’s intake at 2–6 years [44]. These findings are supported by a longitudinal study of older children where frequency of consumption of home-cooked fruit and vegetables at 6 months of age, predicted a higher proportional intake of fruit and vegetables at 7 years [14].

The studies involving exposure to vegetables and fruit in infancy can be quite complex and confusing with attempted crossover exposures and new foods which might be either fruit or vegetables [53]. However, what they show in general is that some vegetables are more difficult than others (green beans versus carrots), with longer exposure periods needed for the more aversive, usually bitter, tastes. On the whole, it can be concluded that it is relatively easy to induce a preference within the usual period of the introduction of complementary foods, that earlier introduction within the time period facilitates acceptance, and the greater the variety of foods introduced, the more likely the infant is to readily accept other foods.

#### Texture

The concept of a sensitive period for the introduction of food of a texture other than puree was first suggested by Illingworth [54] and was based on case studies of hospitalised infants. Past and current research supports this observation, suggesting that it might be easier to get infants to accept new textures, and to progress with texture acceptance, if they are introduced earlier within accepted time frames for introduction. It is usual practice within the UK for pureed food to be offered between the ages 4 and 6 months of age. A survey carried out in the UK in 2011 reported that approximately 80 % of infants had been given their first foods by the age of 5 months [1]. Subsequent to this, it is advised that more ‘lumpy’ solids are to be given from around 6 months of age [55] and over 50 % of infants in one study had been given foods that required chewing by the age of 7 months [56]. The acceptance of a wider range of textures by the end of the first year is important when we consider the onset of neophobia in the second year of life and the type of foods that commonly present with complex and or multiple textures. The ‘mouth feel’ of textured food is difficult for many children and they typically prefer smooth foods to foods with’ bits’ in them [4•]. Most fruit and vegetables, unless pureed, are foods which have complex textures. A tomato, for example, has a firm skin, a pulp and seeds; all of which require different oral-motor skills to process them. These oral-motor skills are usually learnt between the ages of 6 and 12 months, the period in which the tongue learns to move solid food around the mouth in preparation for swallow, and this ability is dependent upon the experience of textured food within the mouth [55], rather than on any particular age or developmental stage.

It has been observed, again in hospitalised infants, that those who are introduced late in the first year to textures other than smooth or puree, are less likely to accept difficult textures in later childhood. Indeed, children who are introduced after the first year are more likely to become orally defensive and refuse any other than a smooth texture. They are more likely to gag and vomit when given solid foods, and in response to this, parents become more reluctant to persevere with solid food introduction [56–58].

There is, however, only one experimental study which looks at texture progression and acceptance in infancy. Twelve-month-old infants were given pureed and chopped carrots; infants consumed more of the pureed carrots, but there was variability in the infants’ willingness to take the chopped carrot. The strongest predictor of the acceptance of chopped carrot at 12 months, other than the presence of teeth, was earlier experienced with textured foods [59]. In addition, children who had been used to a high variety of different foods in their diet ate more of the chopped carrot; this again reflects the generalisation effect, the greater the experience, the greater the willingness to try. A small advantage associated with breastfeeding was observed in these children; longer duration of breastfeeding was associated with higher variety in the diet and greater acceptance of chopped carrot.

Two analyses of longitudinal data bases show a similar advantage of early experience. In the first study [60], children introduced to lumpy solids after the after the age of 10 months were more difficult to feed and were fussier at 15 months than were children introduced earlier to lumpy solids. Those introduced to complementary foods after 10 months also ate fewer family foods and more baby foods such as baby cereals. In a second analysis of these data, children introduced to lumpy solids after the age of 10 months were reported as having more feeding problems at 7 years. They were also reported as eating fewer portions of fruit and vegetables and ate more of all of ten categories of fruit and vegetables assessed at 7 years. Those introduced to complementary foods by 6 months ate more green leafy vegetables, green vegetables, tomatoes and citrus fruits than those who were introduced later, even when breastfeeding duration was controlled for within the analysis [61].

Given that these data are based on longitudinal reports, it could be that those introduced later to lumpy solids were more difficult to feed and more reluctant to accept textured foods.

A further longitudinal questionnaire study [48] did observe a relationship between acceptance of a range of textured foods and feeding style, whether breast fed or formula fed. But interestingly, breastfeeding and bottle feeding with a’ chewing style’ teat were both reported as promoting feeding progress. However, it was also noted that food acceptance was greater where family foods were given more often to the infant. There is then a relationship between longer breastfeeding duration and the extent to which family foods, rather than pureed or commercially available baby foods, are fed to the infant as first foods [62], and this early and prolonged introductory period to real food tastes and textures generally influences subsequent texture acceptance.

#### Sensory Sensitivity

One of the newer areas of interest in food acceptance is that of sensory hypersensitivity or hyper reactivity to sensory arousal. This denotes an over awareness and responsivity to stimuli, an over arousal which can give rise to an aversive reaction to normally non-threatening factors in the environment [63]. Specifically, oral/visual/tactile/olfactory hypersensitivity can lead to a limited range of foods accepted within the diet, a limited acceptance of textures and a fear of trying new foods [58].

It has been found that preschool children who are tactile defensive have more problems with food of various textures [64]; that boys with higher smell reactivity are more neophobic [47•] and that preschool children with taste, smell and tactile sensitivity are more neophobic and less likely to model their mother’s fruit and vegetable consumption [65]. The effects of this sensory sensitivity can also be observed in the food choices of older children; taste/ smell sensitivity was found to be associated with a limited range diet in children from 5 to 10 years [66]. A relationship has also been found between neophobia, or limited acceptance of range, and the hedonic evaluation of tactile substances in children aged 2–4 years [67] and 4–7 years [68].

As this hypersensitivity would seem to be an innate trait, then it might also contribute to the reluctance of some infants in the early introductory period to accept new flavours, or more specifically textures; and, such an interaction has been observed between early experience and infant sensory sensitivity. In infants introduced to complementary foods early or late within the 4- to 6-month period of introduction, and screened using the Dunn Infant Sensory Profile [69], it was found that infant sensory sensitivity predicted consumption of a new food. The higher the sensory reactivity the lower the consumption of a new food taste. In addition, the relationship between tactile hypersensitivity and acceptance of the new food was moderated by the age of introduction to complementary food. Those infants who were introduced later within the 4 to 6 month period were less likely to accept the new food if they were scored highly for sensory reactivity [70].

## Conclusion

A combination of breastfeeding with the timely introduction of complementary foods may confer a generalisation effect on the acceptance of new foods, and would seem the strategy which best predicts the subsequent acceptance of foods such as fruit and vegetables. However, it is clear that whereas breastfeeding is not a necessary prequel to a wide food acceptance, the timely and frequent introduction of complementary foods of differing tastes and textures is.

There are some data which would seem to support the idea of sensitive periods for the introduction of complementary foods according to both taste and texture, and this effect would appear to be more marked for those infants who are sensory hypersensitive. We also know that there are innate differences between children which make some tastes and textures more difficult to accept and that these tastes and textures are those that are associated with vegetables and especially green leafy vegetables.

A generalisation effect has been noticed at all stages; the more variation in tastes and textures that are experienced the more willing the child is to try new foods. This gives rise to the advantage conferred by breastfeeding over formula feeding, but also means that complementary foods should be given with frequent taste variation, and that the early introduction of textured complementary foods (other than smooth puree) confers an advantage on the subsequent acceptance of other more complex textures, such as those found in most fruits and vegetables.

In conclusion, then it would seem that both breastfeeding and the timely introduction of a variety of tastes and food textures would best predict acceptance and subsequent inclusion of a wide range of foods, especially fruit and vegetables, within the child’s diet.
